# Salmagundi

**Published:** 1884-07

**Authors:** 


### SALMAGUNDI.

EDITED BY E. G. BETTY, D.D.S.

### Meeting of College Faculties.

There is to be a meeting of the faculties (or representatives of them) of the various dental colleges of the country, in New York city, August 4th of the current year. This meeting is one of far more importance than the majority of the profession have any idea, and one that will or ought to have a powerful bearing upon the much mooted question of the regulation of the requirements necessary for admission to a dental institution, the length of time of pupilage, etc., etc., not to mention the financial aspect of the matter, so far as fees and expenses to the student are concerned.

It is high time that something in the way of concerted action should be taken upon this most vital point concerning the status of the profession as such, and of the standing of its practitioners individually.

There is at present a great jumble of opinions upon the subject of professional education, no two men, scarcely, agreeing upon any specified standard, much less are there two institutions that treat the matter from the same standpoint. It is imperatively essential to the future growth, prosperity, and standing of the profession, that new members entering it be thoroughly qualified in all respects to assume its responsibilities and discharge them with consummate ability and honor. To this end there *must* be harmony of action among the several colleges, dental departments of universities, etc., so that all shall be required to rise to a specified level before they are permitted to shove out for themselves, some to retrograde, others to progress. If, as we hope, and have some reason to suppose, the profession is to march ahead, we must look to the quality of the men who are intrusted with its honor and advancement. All these things and many others can only obtain when there is national unity of action among the educating centres. The medical colleges of the country, or at least the great majority of the better ones, are united under common laws and rules, formulated by the Association for the guidance of all members, all requirements being the same in all, there also being a standard schedule of fees. This has operated well; in fact, putting more money into the treasury of small colleges by raising the fees, where formerly they depended upon a large number of students to supply their expenses, by holding forth the inducement of *cheap* instruction.

Under such an Association the rivalry between the colleges must be based upon the thoroughness and sterling quality of the instruction, and not upon an underbidding in the matter of fees that has wrought so much damage, because it is demoralizing equally to the instructor, the student, and the profession.

Another point, and one of "great pith and moment," is the urgent necessity of abolishing all State examining boards, regents, or whatsoever they may be, and thus protect the colleges and through them, the profession; also, absolutely demanding graduation as the one great qualification.

With an association of dental colleges and the momentum necessarily accruing to such an influential body, the state legislatures will more likely be induced to pass such laws as suggested.

The result cannot but be of vast influence upon the profession a generation hence, for it will take that length of time to produce a radical change upon the existing order of things.

We hope much from this meeting, and the REGISTER expects to lay its deliberations before its readers, for let the result be great or small, the move is one in the true direction, and if it be not fruitful now, "yet it will be."

---

IT IS now about two years since Dr. Jerry Robinson introduced his textile metallic filling material. During this time it has not only been brought to the notice of the profession pretty generally, but it has apparently won a place for itself among the list of filling materials that is high up among the reliables.

Two years time is scarcely sufficient to judge of its enduring qualities, yet the test of this time gives great promise for the future. With many, it has taken the place of amalgam in about eighty or ninety per cent. of cases; this alone is a good recommendation for it.

We, in common with others, have used it largely for the past couple of years and find, so far as we have yet been able to see, that it returns well for the labor of putting it in. It is not advisable to introduce it in very large pieces, as they cannot be handled with accuracy. The filling may be started with a cylinder or wad, the balance packed in, in the form of strips, such as heavy gold foil is cut into. *Thorough* malleting throughout the whole process of filling is very necessary, for, if not made dense, the filling, especially one on an approximate surface, will disintegrate under the wear of mastication. In preparing cavities on the proximate surfaces of bicuspids and molars, it is best to cut the lateral walls perfectly square, as, if they are flared, it is next to impossible to make the joint perfect, and besides this would leave a very thin portion of the material to guard the enamel.

It adds greatly to the density of these fillings to use a matrix, as the mallet can then be used liberally and without fear of dislodging any portion of the filling. The only objection so far apparent is, that when used in combination with gold and both metals exposed to the fluids of the mouth, the textile material will become black.

This is a slight fault that may be remedied to some extent by not exposing the material in places where it will be seen. Furthermore, the darkening appears to be on the exposed surface and not in the interior of the tooth. If no harm results to the tooth from the discoloration of the metal, it may pass without comment.

---

In addition to the use of opium in its many forms, hasheesh, ether, chloroform, and other drugs used to produce nervous stimulation, it appears that quinine has been added to the list, as the following article clipped from one of several daily papers containing it seems to indicate, and we give it for what it is worth :—

Said a distinguished medical practitioner, who has grown gray in his profession, speaking of the report that the use of quinine as a stimulant is becoming a very common habit among men of business and ladies of society:

"Yes, it is unquestionably true that the great increase in the sales of quinine during the last five years by retail druggists is very largely referable to what may be styled the quinine habit; and it is fully as frequent among women in society as it is with men whose nerves are overtaxed by hard work. And I may say to you, though many will dispute it, that of the two the quinine habit is more rapid in its ravages, when once thoroughly established, more difficult to break, and more dangerous in every respect than the habitual use of opium or its preparations. Few, say practicing physicians, are aware of the tremendous potency of this drug in its effects upon the nervous system. As you know, depending upon the quantity taken, quinine possesses four very distinct properties, being, in very small doses, tonic and nervine; in moderate doses, directly stimulant; in large doses, sedative and soporific; in very large doses, intoxicating, producing a peculiar species of drunkenness, similar in its features to masked epilepsy, in which, while performing customary actions, and talking with the coherence of a person in full possession of his senses, the victim is really perfectly unconscious of what he is doing and totally irresponsible. There is no question that the regular use of the drug as a stimulant is rapidly increasing among the higher classes, and the fact is one of the most lamentable that has come under my notice for years. The way a man gets into it in the first place is very simple. He feels a little unstrung and out of tune, perhaps, and so consults the family physician, who suggests a few doses of quinine. In a day or two he feels singularly improved; his brain is clearer and bright; his physical energies seem to have renewed their youth. Elated with the result, whenever he feels down spirited or out of sorts, he resorts, of course, to the remedy that has once served his purpose so well, and very soon has acquired the habit of using the drug in regular daily doses. In three months, so insidious are its effects, the quinine habit is fully established, and the probability is that the man (or woman, as the case may be) has not five years to live.

Worse still, so peculiar are the effects of the salt on the nervous system, there is a strong probability that the victim will die of suicide, for it is a singular fact that no toxic in the materia medica acts so directly and rapidly to produce suicidal predisposition and impulse. Morphia has no such effect, deplorable as its ravages are. The morphia habit generally transforms the most truthful man or woman into the most inveterate liar in the course of two or three years—a romancer of the wildest type. On the other hand, while quinine produces no perceptible effect on the veracity, it leads to a nervous irritability that is intolerable alike to its victim and his associates, and frequently ends in the sudden development of suicidal mania. Again, a patient may be reduced to the verge of the grave by morphia, and still recover a remnant of physical and nervous energy when the drug has been eliminated from the system; but when once the system gives way under the cumulative influence of quinine, the break-down is irrevocable. In the course of an experience embracing thirty-five cases of the quinine habit in its later stages, during the last two years, I have never seen a case in which the victim was good for anything after the habit was broken, and, as a rule, the patient collapses and dies if the withdrawal of the stimulant is persevered in. Knowing these facts, I cannot tell you how I dread to prescribe quinine to men a little fagged out with overwork, and I think it is time that medical practitioners began to be as cautious with it as they are with morphia."

DETROIT, MICH., June 11, 1884.

EDITOR SALMAGUNDI: *My dear Sally*—You may sally forth and proclaim to the world in general, and the boys in particular, that if they would have nice and soft working foil for the generality of cavities, and more especially the quite large, but not complicated, ones, or those requiring caution, they can have it if they will anneal the gold in the book. Put two or three sheets in the center of an empty book, or as much as you require for immediate use (its better to have it fresh No. 4); then place in the cooking range oven, as hot as you can get the oven, and when the outside corner is pretty thoroughly scorched—done to a nice brown—it may be taken out, prepared, and used without further annealing. But should it be desired to have it more cohesive, let the foil be prepared in the operator's customary way, and laid on a sheet of mica: then place on the top shelf in the oven. This will make it more cohesive than when annealed in the book, but not so sticky as when annealed in the flame of the lamp. I feel satisfied that in the ordinary way of annealing foil it is heated too hot. There is a proper degree of heat at which, when gold is heated or annealed, will give the best results; and that degree, I am sure too, is below the red. What is it? Experimenters, experiment! More anon. Yours, etc.,

E. C. MOORE.

---

### IMPROVEMENTS IN FILE CARRIERS.

CINCINNATI, June 12, 1884.

EDITOR SALMAGUNDI: Take a piece of octagonal steel one-fourth inch thick and five and one-half inches long. Turn a shank on each end one and one-half inches long, in the end of which saw a slit one-sixteenth inch deep, and enlarge with a file so as to admit the end of a broken separating file. After introducing the file, place a small piece of tinners' solder on the back at its junction with the shank, add a drop or two of a saturated solution of zinc chloride, and hold the *shank* over the flame of a spirit lamp or Bunsen burner till the solder flows, when it should be removed and allowed to cool. This carrier is easily controlled and is superor to an unbroken file. It is well to put a different size in each end. They may be placed at any angle, or may be straight. If the file should break, the shank should be reheated, and when the solder flows, the stump can be removed and another piece soldered in. Respectfully,

WILLIAM D. KEMPTON.

---

"DR. FARR, an English scientist, says that if one could watch the march of one million through life, the following result would be observable: Nearly 150,000 will die the first year, 53,000 the second year, 28,000 the third year, and less than 4,000 in the thirteenth year. At the end of forty-five years 500,000 will have died. At the end of sixty years 370,000 will be still living; at the end of eighty years, 97,000; at eighty-five years, 31,000; and at ninety-five years, 2,100. At the end of one hundred years there will be 223, and at the end of 108 years there will be but one survivor."

---

THE hot weather is now upon us, making us (as old Sol did the traveler in the fable) doff our winter garb (and habits), and betake ourselves to the cool, green shade of the rural districts, where we may in peace listen to the low of the kine and cackle of the hen, while we meditate upon the uncertainties of life in general, and the practice of dentistry in particular. Meanwhile the "bugs" are slaughtering teeth in calm contentment, being conscious that "While the cat's away the mice may play."

---

LITTLE son of a bill broker to his papa: "Papa, what is business honor?" "Business honor? Well, Alphonso, my boy, suppose one of our customers sends 1,000 francs too much." "Yes, papa." "Well, I give Mr .Radipeau, my partner, 500 of it." Voila tout.—*Rehoboth Sunday Herald.*

---

THE German Government has distributed large sums of money to several of her scientific men for their labors in Egypt during the cholera epidemic to discover its cause. Dr. Koch, the discoverer of tubercle bacillus, received 150,000 marks.

THE annual announcements of the Pennsylvania College of Dental Surgery, and the Dental Department of the University of Maryland, for the sessions of 1884-5, are at hand, giving assurance of prosperity for the future.

---

"THE late Sam. Ward said anything green could be made into a salad. The young medical graduate may therefore be said to be in his salad season. This may with equal propriety be applied to the young dental graduate."

---

ERIC E. SATTLER, M. D. of Cincinnati, author of "The History of Tuberculosis," has been appointed Demonstrator of Anatomy in the Miami Medical College, *vice* Prof. W. H. Kearney resigned.

---

"MILKMAN, milkman, tell me true: do you put in indigo to make your milk so blue?" "Nay," quoth the poetic milkman, "indeed I say no lie: 'tis the reflection in the water of the pale blue sky."

---

Now that M. Pasteur has found a cure for hydrophobia by inoculation, he ought to turn his attention to the man who spits. This disgusting disease is alarmingly common.—*New York Tribune.*

---

DR. O. N. HEISE, of Cincinnati, was married to Miss Amelia Marqua, of the same city, June 25th. We wish the young couple every joy, and uninterrupted prosperity.

